# Association between single nucleotide polymorphism of human angiotensin-converting enzyme 2 gene locus and clinical severity of COVID-19

**DOI:** 10.1186/s43042-022-00331-8

**Published:** 2022-08-23

**Authors:** Shaimaa A. Elbadri, Nermeen M. A. Abdallah, Mona El-Shokry, Amr Gaber, Mahmoud Kh. Elsayed

**Affiliations:** 1 Medical Microbiology and Immunology, Faculty of Medicine, Ain Shams University, Cairo, Egypt; 2 Anesthesia, Intensive Care and Pain Management, Faculty of Medicine, Ain Shams University, Cairo, Egypt

**Keywords:** COVID-19, SARS-CoV-2, ACE2, SNP, Severity of COVID-19

## Abstract

**Background:**

Coronavirus disease 2019 (COVID-19) is a devastating pandemic-causing disease with a variable severity among populations. Genetic studies have pinpointed angiotensin-converting enzyme 2 (ACE2), a key enzyme for viral entry, for its possible linkage to the disease progression. The present study aimed to investigate the potential association between single nucleotide polymorphisms (SNPs) of human ACE2 gene with the severity and outcomes of COVID-19 for better patient management.

**Methods:**

In this observational cross-sectional study, COVID-19 confirmed patients were classified into moderate and severe cases according to the “Ain Shams University Hospitals Pocket Guide for COVID-19 Diagnosis.” Genetic analysis of ACE2 SNP rs2048683 was carried out using a TaqMan assay with the real-time polymerase chain reaction (PCR) technique.

**Results:**

Among 90 confirmed COVID-19 patients, 78.9% (71/90) were classified as severe, and 21.1% (19/90) were classified as moderate. Laboratory biomarkers were significantly (*P* = 0.000) higher in the severe group than in the moderate group. Similarly, associated comorbidities such as hypertension were significant (*P* = 0.000) in the severe group, whereas asthma and deep venous thrombosis were significant in the moderate group (*P* = 0.007 and 0.006, respectively). Elevated serum ferritin level (odds ratio (OR) 162.589, 95% confidence interval (CI) 8.108–3260.293) and ACE2 rs2048683 genotype GG/G (OR 5.852, 95% CI 1.586–21.591) were both considered independent risk factors for severe disease.

**Conclusion:**

The findings of the present study provide preliminary evidence of an association between ACE2 rs2048683 SNPs and COVID-19 severity in the Egyptian population, which may inform the need for targeted management.

**Supplementary Information:**

The online version contains supplementary material available at 10.1186/s43042-022-00331-8.

## Introduction

Severe acute respiratory syndrome coronavirus 2 (SARS-CoV-2) a human coronavirus that causes coronavirus disease 2019 (COVID-19) was declared a pandemic in March 2020 [1] and was mostly associated with respiratory symptoms. Nevertheless, extrapulmonary presentations have been documented as well [2].

Viral entry into the cell is mediated through interaction between spike protein projections at the viral surface and angiotensin-converting enzyme 2 (ACE2). This interaction is facilitated by cellular proteases such as transmembrane protease serine 2 [3].

ACE2 is a type 1 transmembrane enzyme expressed in a wide variety of cells in the body such as corneal, intestinal, and respiratory tract cells. It has been long known for its role in the renin-angiotensin pathway. ACE2 is considered to have a cardio-pulmonary protective effects due to its role in controlling the metabolism of angiotensin II and, to a lesser extent, angiotensin I by increasing vasodilator angiotensin 1–7 and decreasing angiotensin II levels [4]. Downregulation of ACE2 by endocytosis after viral entry releases ACE and angiotensin II leading to increased vascular permeability and subsequent lung injury [5].

The role of ACE2 in the pathogenesis of COVID-19 is complex; the overall picture of COVID-19 progression is the result of the interaction of multifactorial aspects such as ACE2 expression site, level of expression and virus binding affinity [6]. Given that the ACE2 gene is X linked (locus Xp22.2), males are hemizygous for the gene [7]. Variations in the ACE2 gene may affect the receptor structure, resulting in modulating the affinity to the virus, or disrupt the proteolytic cleavage of ACE2, thereby affecting the level of soluble ACE2, or the non-coding sequence of ACE2 [8]. The majority of ACE2 single nucleotide polymorphisms (SNPs) are introns, which do not affect the receptor protein structure but do affect its expression level [9].

ACE2 expression in lung epithelial cells increases over time after SARS-CoV-2 infection and remains at a high level for 48 h post-infection, showing that the role of ACE2 is not related only to susceptibility to infection, but also to post-infection regulation and immune response to this virus [10]

Since the start of the pandemic, researchers have been searching for a possible explanation for the variation in susceptibility to infection, disease course, and vulnerability among SARS-CoV-2-infected patients. Old age and associated comorbidities were thought to be important risk factors. However, similar disease deterioration was experienced among healthy and young groups [11]. Blood group, gender, race, ethnicity, viral load, and environmental factors were also linked to the variation in host responses. Additionally, genetic factors affecting SARS-CoV-2 entry and immune response have been investigated for possible associations [11, 12].

Cardiovascular and metabolic disorders are potentially associated with the rs2048683 SNP [13] [14]. Similar linkages between the same SNP with COVID-19 severity and the need for hospitalization have been studied in 61 Caucasian COVID-19 patients by Wooster and colleagues [15].

The above-mentioned SNP is considered an intronic variation in chrX:15590376 (GRCh38.p13), located between exon 14 and 15 of the ACE2 gene. Its global alternative allele frequency is *G* = 0.638852 (96251/266514, ALFA Project) and its frequency in African populations is *G* = 0.7731. The highest alternative allele frequency was identified in East Asian populations, while the lowest frequency was identified in European populations [16]

To our knowledge, this study is the first to investigate the rs2048683 SNP and its association with severity and outcomes among Egyptian COVID-19 patients. This could help researchers to better understand genetic predisposition to SARS-CoV-2 infection to identify patients at risk and determine the best treatment options.

## Patients and methods

This observational cross-sectional study was conducted on 90 clinically and PCR confirmed-COVID-19 patients admitted to the isolation ward of Ain Shams University (ASU) Hospital between October 2020 and March 2021. This study was approved by the Ethical Committee of the Faculty of Medicine at Ain Shams University (FMASU M D 237/2020). Informed consent was obtained from each patient or patient's next of kin.

### Data collection

Data were collected from medical records and included: sociodemographic data, clinical presentation on admission, and laboratory and radiological findings.

### Classification of admitted patients

Based on clinical, laboratory and radiological findings, admitted patients were classified into mild, moderate, and severe cases according to the “Ain Shams University Hospitals Pocket Guide for COVID-19 Diagnosis” (Table 1, Additional file 1).

**Table 1:** Classification of laboratory and clinically confirmed COVID-19 patients

Classification	COVID-19 cases
Mild cases	A) Asymptomatic with/without abnormal laboratory or HRCT findings of COVID-19 pneumonia B) Symptomatic with no HRCT finding of COVID-19 pneumonia with/without risk factors*
Moderate cases	Clinical signs of non-severe pneumonia (i.e., fever, cough, dyspnea) and HRCT findings of COVID-19 pneumonia and/or abnormal laboratory findings
Severe to critically severe cases	A) Clinical signs of severe pneumonia (e.g., respiratory rate > 30 breath/min, severe respiratory distress or SpO_2_ < 93% on room air and HRCT finding of COVID-19 pneumonia
	B) Occurrence of respiratory failure requiring mechanical ventilation, shock, extrapulmonary organ failure, and treatment in ICU

HRCT, High-Resolution Computed Tomography; ICU, intensive care unit. Risk factor*, age > 60 years, body mass index (BMI) > 40, pregnancy, comorbidities such as hypertension, diabetes, cardiovascular disease, chronic respiratory disease (asthma, chronic obstructive pulmonary disease (COPD)), chronic kidney disease, active malignancy or immunosuppressive disease or drugs

### Molecular detection of the rs2048683 SNP

Ethylenediaminetetraacetic acid (EDTA)-anticoagulated 3 ml blood samples were collected under complete aseptic conditions from each participant and were stored at -80Cº till real-time PCR processing.

Human DNA isolation and purification were performed with Thermo Scientific™ Gene JET Whole Blood Genomic DNA Purification Mini Kits #K0781 (Thermo Scientific, USA) according to manufacturer guidelines. Briefly, proteinase K and lysis solution were added to whole blood samples in a sterile microcentrifuge tube, then incubated and mixed with ethanol thoroughly. The prepared mixture was transferred to the Gene JET™ spin column and centrifuged for 1 min at 6000×*g*. Then washing was done in 2 steps using washing buffer followed by centrifugation. After that, elution buffer was added to the column membrane to elute genomic DNA.

The region flanking the rs2048683 SNP was amplified using sequence-specific primers to detect target SNP with two allele-specific TaqMan^®^ Minor groove binder probes. One probe was labeled with fluorescein amidite (FAM) dye that detected the T allele and the other probe was labeled with Victoria (VIC) dye that detected the G sequence allele. The context sequence was [VIC/FAM]TGGCAGTGTAGATATCTTTATGAAG[G/T]GGTAATTTCATCTAATTTAGGTATA. The 25 μL reaction mixture contained: the TaqMan Genotyping Master Mix (Catalog number: 4371353, (Applied Biosystems, USA) and 5´nuclease Predesigned TaqMan^®^ SNP Genotyping Assay (Catalog number: 4351379, Applied Biosystems, USA) was mixed with 20 ng of DNA. Real-time PCR was performed according to the manufacturer’s protocols as follows: hold cycle at 95 °C for 10 min, 45 cycles (at 95 °C for 15 s for denaturation and at 60 °C for 1 min for primer annealing/extension).

SNP analysis and allelic discrimination were automatically obtained from Rotor-Gene Q software version 2.3.3.5.

### Statistical data analysis

Generated data were analyzed using the Statistical Package for Social Sciences (SPSS) version 20 (USA). Continuous data are presented as means and standard deviations (± SDs) and were compared between the two groups (moderate and severe) using an unpaired t test. Nonparametric data are presented as mediums and interquartile ranges (± IQRs) and were compared between the two groups using the Mann–Whitney *U *test. Dichotomous data are presented as frequency numbers and percentages and were compared between the two groups using the Chi-square test. A *P* value ≤ 0.05 was considered significant.

Univariate logistic regression analyses were used to test the effect of unevenly distributed variables in the two groups on the severity of the disease. Multivariate logistic regression analysis was used for the resulted significant data to determine the independent factors associated with disease severity.

## Results

The majority (71.1%, 64/90) of the patients enrolled in this study were male. Participants’ ages ranged from 19 to 91 years, and the mean age was 61.57 years (± 13.55 SDs). Most (85.5%, 77/90) participants resided in Cairo governorate and the remaining resided in Giza and Qalyoubia governorates (7.25% each). Most (78.9%, 71/90) of the patients were categorized as severe cases, while (21.1%, 19/90) were categorized as moderate cases according to the ASU pocket guide. No statistically significant difference was identified between the moderate and severe groups.

**Fig. 1:**
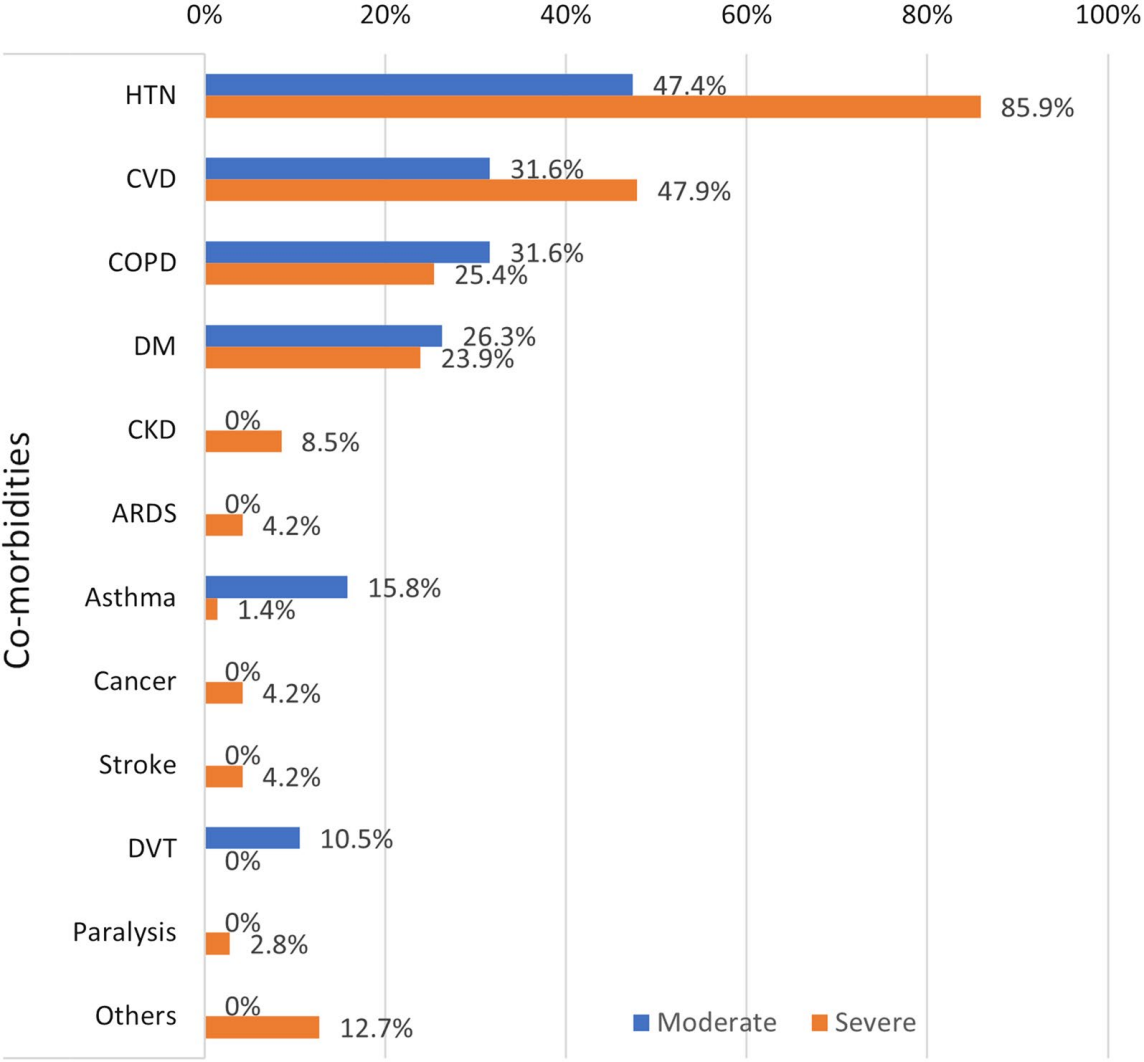
Different comorbidities among the studied patients’ groups

Regarding the associated comorbidities, hypertension was significant (*P* = 0.000) in the severe group, whereas asthma and deep venous thrombosis (DVT) were significant in the moderate group (*P* = 0.007 and 0.006, respectively) (Fig. 1).

Lymphopenia was significant among patients in the severe group (88.7%, 63/71) compared to (52.6%, 10/19) in the moderate group (*P* = 0.000). Levels of C-reactive protein (CRP), serum ferritin, d-dimer, serum creatinine, and liver enzymes were significantly elevated in the severe group compared to the moderate group (*P* = 0.000) (Additional file 2: Table S1).

The classification of patients’ outcomes highlighted that the majority (88.9%, 80/90) of enrolled patients improved. According to the patients’ groups, all 19 moderate cases recovered completely and were discharged, and (85.9%, 61/71) of severe cases showed improvement. In contrast, (14.1%, 10/71) of patients with severe disease passed away.

### Genotype and allele frequencies of the ACE2 SNP

rs2048683 genotypes and allele frequencies (major G allele and minor T allele frequencies) were assessed in relation to disease severity, associated comorbidities, and outcome of the patients.

The GG/G genotypes were significantly more prevalent in the severe group (78.9%, 56/71), whereas TT/T genotypes were more prevalent in the moderate group (57.9%, 11/19) (Table 2).

**Table 2:** Genotype distribution and allele frequencies of rs2048683 among the studied patients and their associations with clinical diseases

	All patients n = 90 F:26, M:64	Moderate n = 19 F:5, M:14	Severe n = 71 F:21, M:50	P-value*	Hypertension n = 70	Non hypertensive	P-value*	CVD n = 40		P-value*	DM n = 22		P-value*
Gene allele*													
G	82 (70.7%)	8 (33.3%)	74 (80.4%)	**0.000**	73 (79.3%)	9 (37.5%)	**0.000**	33 (70.2%)	49 (71.0%)	0.924	22 (81.5%)	60 (67.4%)	0.160
T	34 (29.3%)	16 (66.7%)	18 (19.6%)		19 (20.7%)	15 (62.5%)		14 (29.8%)	20 (29.0%)		5 (18.5%)	29 (32.6%)	
*rs2048683 genotype*													
GG/G	59 (65.6%)	3 (15.8%)	56 (78.9%)	**0.000**	53 (75.7%)	6 (30.0%)	**0.001**	28 (70.0%)	31 (62.0%)	**0.047**	17 (77.3%)	42 (61.8%)	0.282
GT	7 (7.8%)	5 (26.3%)	2 (2.8%)		4 (5.7%)	3 (15.0%)		0 (0.0%)	7 (14.0%)		2 (9.1%)	5 (7.4%)	
TT/T	24 (26.7%)	11 (57.9%)	13 (18.3%)		13 (18.6%)	11 (55.0%)		12 (30.0%)	12 (24.0%)		3 (13.6%)	21 (30.9%)	
*rs2048683 genotype in females*													
GG	16 (17.8%)	0 (0.0%)	16 (22.5%)	**0.000**	16 (22.9%)	0 (0.0%)	**0.021**	5 (12.5%)	11 (22.0%)	**0.077**	3 (13.6%)	13 (19.1%)	0.574
GT	7 (7.8%)	5 (26.3%)	2 (2.8%)		4 (5.7%)	3 (15.0%)		0 (0.0%)	7 (14.0%)		2 (9.1%)	5 (7.4%)	
TT	3 (3.3%)	0 (0.0%)	3 (4.2%)		2 (2.9%)	1 (5.0%)		2 (5.0%)	1 (2.0%)		0 (0.0%)	3 (4.4%)	
*rs2048683 genotype in males*													
G	43 (47.8%)	3 (15.8%)	40 (56.3%)	**0.000**	37 (52.9%)	6 (30.0%)	**0.003**	23 (57.5%)	20 (40.0%)	0.659	14 (63.6%)	29 (42.6%)	0.120
T	21 (23.3%)	11 (57.9%)	10 (14.1%)		11 (15.7%)	10 (50.0%)		10 (25.0%)	11 (22.0%)		3 (13.6%)	18 (26.5%)	

G allele: male G, female GG + GT; T allele: male T, female TT + GT

Bold indicates significance

*Chi-square test; the G allele is represented twice among females and only once among males, therefore, the total count of G alleles among all patients is 116, calculated as follows: (5 × 2 + 14) + (21 × 2 + 50) in the severe and moderate groups, respectively

Regarding allelic assessment, out of 116 alleles, the G allele was the major (70.7%, 82/116) allele detected among all patients compared to the T allele, which was detected among (29.3%, 34/116) of patients.

A statistically significant difference was found between allelic frequencies and disease severity. Out of 92 alleles in the severe group, the G allele was detected in (80.4%, 74/92), which was significantly more than the T allele. In contrast, out of 24 alleles in the moderate group, the T allele was detected in (66.7%, 16/24) and was significantly more than the G allele (*P* = 0.000) (Table 2).

**Fig. 2:**
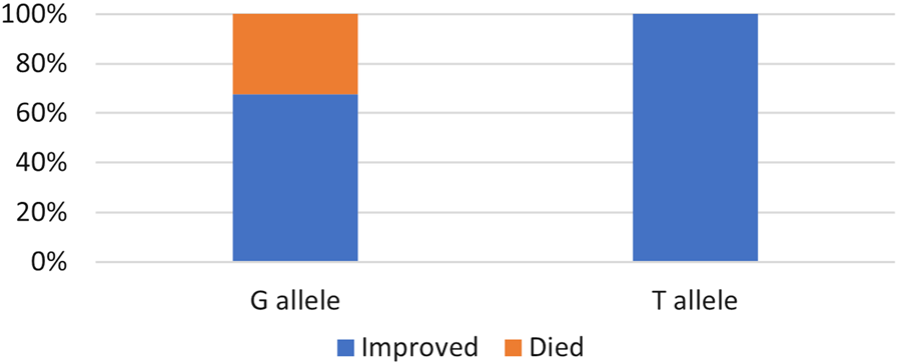
Association of ACE2 SNP with the disease outcome

There was a significant difference between the studied gene alleles and disease outcome, with all deceased patients having the G allele (*P* = 0.025) (Fig. 2).

Univariate logistic regression analysis was performed on variables that were unequally distributed among the different groups to connect them with the disease severity. Then multivariate logistic regression analysis was done revealed that both elevated serum ferritin levels (OR 162.589, 95% CI 8.108–3260.293) and ACE2 rs2048683 genotypes GG/G (OR 5.852, 95% CI 1.586–21.591) were independently associated with disease severity (Additional file 2: Table S2a, b).

## Discussion

This study confirms the association of ACE2 SNPs and epigenetic factors with the severity of SARS-COV-2 infection. Male predominance (71%) in the study can be attributed to associated risk factors that are culturally linked to males, such as smoking [17]. Furthermore, females have stronger immune responses than males, either because of direct effects of sex hormones or an underlying discrepancy in the expression of genes encoded on the X and Y chromosomes [17, 18].

We identified that patients in the severe group had significantly higher levels of laboratory biomarkers (CRP, serum ferritin, and D-dimer) compared to patients in the moderate group. This finding is consistent with that reported in other studies [19, 20]. Given that, elevated biomarkers can predict respiratory deterioration; they are currently considered markers of poor outcome for COVID-19 patients and caused by associated systemic hyperinflammatory reactions [21]. A significant finding of lymphopenia observed among patients was similar to findings in previous studies [10, 19] and can be explained by a direct result of viral invasion or distorted inflammatory cytokines leading to lymphocytes apoptosis [19, 22].

In this study, an elevated serum ferritin level was identified as the only laboratory marker that served as a significant independent risk factor for severe COVID-19. This finding comes in agreement with other studies that linked elevated serum ferritin levels to disease severity and outcome, making it a reliable parameter for predicting poor prognosis and prioritizing patients [23, 24]

Significant differences were observed between ACE2 rs2048683 SNP genotypes and allele frequencies in relation to disease severity and outcome. The proportion of the G allele (major allele) detected in (70.7%) of all patients was significantly higher in the severe group than in the moderate group (*P* = 0.000). This allele frequency (AF) is comparable to that reported in the 1KGP (1000 Genomes Project) from Africa which is 0.8066 and 0.768 in International HapMap Project [16]. Additionally, our results indicate that having ACE2 rs2048983 SNP GG/G genotypes was an independent risk factor for severe COVID-19 disease (OR 5.852).

The observed significant associations between rs2048683 genotypes and COVID-19 severity are in concordance with the findings of Wooster et al., who found that the rs2048683 SNP was one of five SNPs (rs4240157, rs6632680, rs4830965, rs1476524, and rs2048683) that were associated with severe disease and a higher hospitalization rate among Caucasian COVID-19 patients [15]. However, the rs2048683 SNP was not associated with disease severity in two cohorts of British and Chinese COVID-19 patients [25, 26]. In contrast, Martinez-Sanz et al. noticed no increase in susceptibility to SARS-CoV-2 infection associated with the rs2048683 SNP [27]*.*

The ACE2 untranslated gene region and intron polymorphisms such as rs2048683 do not affect ACE2 amino acid and protein structure but may alter mRNA splicing and stability, affecting gene expression and protein levels. As a result, they have the potential to influence disease progression and course [28].

Another previously studied intronic ACE2 SNP, rs2285666, which is located near intron 3 and affects the splicing mechanism, has been linked to a lower infection rate and case fatality rate among Indian individuals [29]. Interestingly, the same rs2285666 SNP was significant among Egyptian patients with severe COVID-19 [30]*.* The differences in AFs of ACE2 gene variants between populations may explain the diverse genetic basis that alters ACE2 functions or expression, thus affecting disease severity and outcomes.

The most common comorbidity identified in the current study was hypertension (HTN). Severe cases showed more comorbidities with a significant statistical difference recorded in HTN (*P* = 0.000). This is consistent with the findings of Guan et al. and Li et al. regarding COVID-19 patients in China [19] [31].

We found a significant association between rs2048683 genotypes and HTN, cardiovascular diseases (*P* = 0.001 and 0.047, respectively). The G allele was significantly more frequent among hypertensive patients (*P* = 0.000). Similarly, Hamet et al. showed that British COVID-19 patients with severe outcomes had a higher percentage of hypertension and diabetes than moderate patients. They investigated the association of 12 ACE2 SNPs with hypertension and discovered that the rs2048683 and rs4646156 SNPs were significantly associated with hypertension [25]*.*

In China, the rs2048683 SNP was associated with an increased risk of developing type 2 diabetes and diabetic-related cardiovascular risk as well as an increased left ventricular mass index, which is an indicator of hypertensive cardiomyopathy [13]. Among Mexican individuals, a link was found between diastolic and systolic hypertension and the rs2048683 SNP in study participants with healthy lipid profiles [14]. Other studies, however, found no link between the rs2048683 SNP and essential hypertension, dyslipidemia, or ischemic stroke [32, 33]*.*

The role of ACE2 variants in cardiovascular and metabolic diseases may be due to decreased ACE2 amount and expression, which increases inflammation and tissue injuries caused by unmetabolized angiotensin II and an unbalanced renin–angiotensin–aldosterone system.

## Conclusion

To the best of our knowledge, this is the first study to look at the genetic link between the ACE2 SNP rs2048683 and disease severity among patients with confirmed COVID-19 in Egypt. We found a possible association between the ACE2 rs2048683 polymorphism and disease progression. Large-scale studies are needed to validate this SNP’s role as a possible genetic marker for disease severity.

## Limitations

The sample size used in this study was relatively small due to high costs associated with molecular testing. We were also challenged with a lack of studies that addressed similar SNPs.

## Supplementary Information

**Additional file 1**. Ain Shams University Hospitals Pocket Guide For COVID-19 Diagnosis.

**Additional file 2**. **Table S1:** Laboratory data among studied patients’ groups. **Table S2a:** Univariate logistic regression analysis for predictors of disease severity. **Table S2b:** Multivariate logistic regression analysis (Backward: Wald) for predictors of classification.

## Data Availability

The datasets used and/or analyzed during the current study are available from the corresponding author on reasonable request.

## Abbreviations

ACE2: Angiotensin-converting enzyme 2
SARS-CoV-2: Severe acute respiratory syndrome coronavirus 2
SNP: Single nucleotide polymorphisms
COVID-19: Coronavirus disease 2019
PCR: Polymerase chain reaction
OR: Odds ratio
CI: Confidence interval
HRCT: High-resolution computed tomography
ICU: Intensive care unit
BMI: Body mass index
HTN: Hypertension
CVD: Cardiovascular disease
COPD: Chronic obstructive pulmonary disease
DM: Diabetes mellitus
CKD: Chronic kidney disease
ARDs: Acute respiratory diseases
DVT: Deep venous thrombosis
EDTA: Ethylenediaminetetraacetic acid
FAM: Fluorescein amidite
VIC: Victoria
CRP: C-reactive protein
