# Supplementary material for: Association between single nucleotide polymorphism of human angiotensin-converting enzyme 2 gene locus and clinical severity of COVID-19
Source: Egypt J Med Hum Genet. 2022 Aug 23;23(1):125. doi: 10.1186/s43042-022-00331-8 (PMC9395935; doi:10.1186/s43042-022-00331-8)
Supplement: Supplementary file 1 — Additional file 1. Ain Shams University Hospitals Pocket Guide For COVID-19 Diagnosis. [file 43042_2022_331_MOESM1_ESM.pdf]

Ain Shams University Hospitals COVID-19 Pocket Guide

Ain Shams University Hospitals COVID-19 Suspected Cases Algorithm

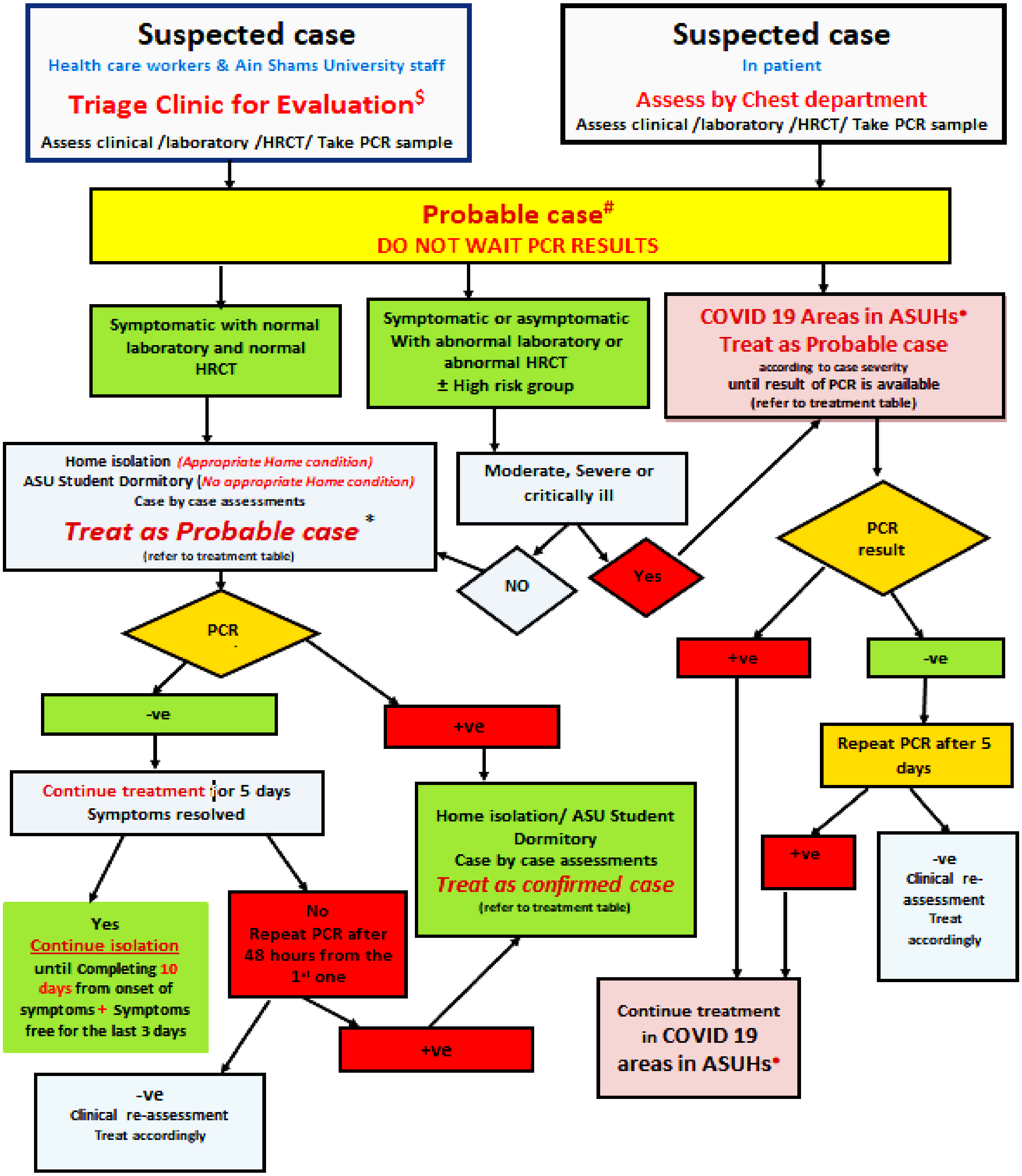

$From 8 am to 8pm covered by Family Medicine / From 8 pm to 8am covered by Internal Medicine in ER→ Responsible to evaluate case and confirm the transfer of the case to site of care

IF THE PATIENT'S CONDITION DETERRIOATES at any time during treatment admit in COVID 19 area* .

#Clinical and radiological picture compatible with CVOID19 infection awaiting PCR result or repeatedly Negative PCR tests collected from different sites with no microbiological evidence of another Infectious etiology

•COVID 19 Areas in ASUHs: Obour hospital, Geriatric hospital, Field hospital and other areas (ER-ICU, old ER, Female Ophthalmology

Treatment Summary

Treatment of Confirmed Case of COVID-19

Mild Cases

	Asymptomatic		Symptomatic	
**Presentation**	**NO abnormal lab findings or HRCT findings of COVID 19 pneumonia**	**Abnormal lab[$] findings or HRCT findings of COVID 19 pneumonia**	**No HRCT findings of COVID 19 pneumonia without risk factors**	**No HRCT findings of COVID 19 pneumonia with risk factors[°]:**
**Site of care**	**Home isolation/ ASU Student Dormitory**	**Designated Hospital or Home isolation/ ASU Student Dormitory ***	**Home isolation Or ASU Student Dormitory**	**Home isolation/ ASU Student Dormitory or Designated hospital ***
**Treatment**	Isolation, rest, good nutrition, good oral hydration & follow up patient response			
		**Treat as Moderate illness**	• **Symptomatic treatment^**<br>• **Supplements#**<br>• **Azithromycin: 500 mg per day for 5days OR Doxycycline oral 200mg once followed by 100mg daily for 5 days**<br>• **Anticoagulation~:** ***Enoxaparin 40 mg/24hr or Rivaroxapan 10mg PO QD or Apixaban 2.5mg PO BID***<br>• **Hydroxychloroquine! 400 mg PO BID for one day followed by 200 mg BID for5-10 days OR Chloroquine phosphate! PO 500 mg BID for 5-10days**	
				**●Available Antiviral drug[⊥]**

Moderate Cases

**Presentation**	**Clinical signs of Non severe pneumonia** (e.g. fever, cough, dyspnea) **& HRCT findings of COVID 19 pneumonia & or Abnormal lab[$]**
**Site of care:**	**Designated Hospital**
**Treatment**	• **Isolation, rest, good nutrition, good oral hydration & follow up patient response**<br>• **Symptomatic treatment^**<br>• **Supplements#**

	• **Azithromycin: 500 mg per day for 5days**<br>• **Anticoagulation~:**<br>○ Enoxaparin 1mg/kg/12hr or Rivaroxapan15mg BID or Apixaban 10 mg PO BID x 7days then 5mg BID<br>• **Hydroxychloroquine! 400 mg PO BID for one day followed by 200 mg BID for5-10 days OR Chloroquine phosphate! PO 500 mg BID for 5-10days**<br>• **Available Antiviral drug**[⊥]<br>• **±Third generation cephalosporin (Ceftriaxone 1 gm /12 hrs IV)**
**Severe Cases**	
**Presentation**	**Clinical signs of Severe pneumonia** (e.g. Respiratory rate > 30 breaths/min; severe respiratory distress; or SpO_2 < 93% on room air) **& HRCT findings of COVID 19 pneumonia**
**Site of care:**	**Designated Hospital/Intermediate care**
**Treatment**	• **Isolation, rest, good nutrition, good oral hydration & follow up patient response**<br>• **Symptomatic treatment^**<br>• **Supplements#**<br>• **Azithromycin: 500 mg per day for 5days**<br>• **Anticoagulation~:**<br>○ Enoxaparin 1mg/kg/12hr<br>• **Hydroxychloroquine! 400 mg PO BID for one day followed by 200 mg BID for5-10 days OR Chloroquine phosphate! PO 500 mg BID for 5-10days**<br>• **Available Antiviral drug**[⊥]<br>• **Prone positioning** should be encouraged (unless contraindicated) (30 min / 2 hours) or as tolerated<br>• **Third generation cephalosporin (Ceftrioxone 1 gm /12 hrs IV)**<br>• **In case of cytokine storm consider adding Tocilizumab (Anti-IL6): 4-8 mg /kg. Dilute in 100 ml of 0.9 % saline and infused over 60 minutes – First dose is calculated as 8 mg/kg and the response is assessed, if the patient needs the second dose it should be calculated as 4 mg/kg after 12 hrs.**<br>• **If Tocilizumab is not available we may give methylpredisonolone (1 mg/kg/day IV for 5 days; then 0.5 mg/kg/day IV for 2 days).**<br>• **Oxygen therapy & further ICU management**
**Critical Cases**	
**Presentation**	Occurrence of respiratory failure requiring mechanical ventilation; Presence of shock; Sepsis, other organ failure that requires monitoring and treatment in the ICU
**Site of care:**	**Designated Hospital/ ICU**
**Treatment**	• **Isolation, rest, good nutrition, good oral hydration & follow up patient response**<br>• **Symptomatic treatment^**<br>• **Supplements#**<br>• **Azithromycin: 500 mg per day for 5days**<br>• **Anticoagulation:** Enoxaparin 1mg/kg/12hr<br>• **Hydroxychloroquine! 400 mg PO BID for one day followed by 200 mg BID for5-10 days OR Chloroquine phosphate! PO 500 mg BID for 5-10days**<br>• **Available Antiviral drug**[⊥]<br>• **Prone positioning** should be encouraged (unless contraindicated) (30 min / 2 hours) or as tolerated

	• **Meropenem** 1gm q 8h **+ Vancomycin** 15-20 mg/kg/dose q 8 to 12 hours **(or Linezolid** 600 mg IV q 12h) **until culture results are available**<br>• **In case of cytokine storm consider adding Tocilizumab (Anti-IL6): 4-8 mg /kg. Dilute in 100 ml of 0.9 % saline and infused over 60 minutes – First dose is calculated as 8 mg/kg and the response is assessed, if the patient needs the second dose it should be calculated as 4 mg/kg after 12 hrs.**<br>• **If Tocilizumab is not available we may give methylpredisonolone (1 mg/kg/day IV for 5 days; then 0.5 mg/kg/day IV for 2 days).**<br>• **Convalescent Plasma if available**<br>• **Oxygen therapy / Non-Invasive ventilation / High-flow nasal cannula /Invasive mechanical ventilation & further ICU management**

Remarks:

- ASU: Ain Shams University Student Dormitory
- *Case by case assessment
- $ Treat at home if D-dimer <1mg/L, Absolute lymphopenia< 800 /µl , Ferritin <500 ng/ml., liver function within normal otherwise treat at Designated hospital
- °: High-risk group:Age above 60 years old, Obesity (BMI>40), pregnancy, comorbidities e.g. cardiovascular disease, diabetes, hypertension, chronic kidney disease or chronic respiratory disease (Asthma, COPD)…etc, Immunosuppressive diseases or drugs or active Malignancy
- **~ If D-dimer ≥ 1 mg/L** consider therapeutic anticoagulation
- **! Baseline ECG & follow up whenever needed** (Contraindicated when QTc > than 450 ms in males and 470 ms in females, Myasthenia gravis, Retinal damage, Epilepsy, G6PD deficiency, Chronic Heart, Kidney or Liver disease & Arrythmias. Maximum duration 5 days in all types of disease categories in outside hospital setting or where close cardiac ECG monitoring cannot be possible.
- ⊥ **Antiviral drug:** e.g. Lopinavir-Ritonavir (200/ 50mg) 2 tablets bid for 5-10 days. Oseltamivir is a neuraminidase inhibitor an enzyme not found on coronaviruses. Therefore, no activity is expected.
- **If patient's condition deteriorates, upgrade level of care**, with immediate arrangement for transfer to hospital
- **^Symptomatic treatment**

Treatment of Probable Case of COVID 19 + Symptoms	
**Presentation**	Clinical and radiological picture compatible with CVOID19 infection awaiting PCR result or repeatedly Negative PCR tests collected from different sites with no microbiological evidence of another Infectious etiology
**Site of care:**	Home isolation or hospital *
**Treatment**	• Managed as the confirmed cases in time of pandemic until PCR result is available<br>• Case management should follow COVID 19 severity stratification (mild, moderate, severe, or critical)<br>DO NOT DELAY TREATMENT

- Fever and myalgia: Paracetamol 500 mg PRN or up to 2 gm/day-
- GIT symptoms: Motility regulator (e.g. Mosapride 2.5.mg 1x3 half an hour before meals) + PPI (Omeperazole 40 mg 1x1 half an hour before breakfast).

- **#Supplements:**

- Vitamin C 1 gm/ day.
- Zinc 50 mg per day.

Table1: RT-PCR timeline:

**Home isolation or Student dormitory isolation**	**RT-PCR is required at the end of the isolation period if not available Continue isolation until Completing 10 days from onset of symptoms + Symptoms free for the last 3 days without medication**<br><br>**NB: Cough and anosmia may be the only persistent symptoms left**
**Designated hospital isolation**	RT-PCR is required after 5 days of treatment<br>• If negative repeat after 24 hrs if still negative; stop treatment and continue isolation for 10 days.<br>• If positive repeat every 48 hrs.

Table 2: Investigations schedule:

Severity	Laboratory	Radiology	ECG
**Mild cases**	CBC, CRP, D-dimer, Ferritin, Liver function tests (ALT – AST) and kidney function tests (S.cr- BUN).<br><br>***Base line, not required to be repeated unless there is symptomatic progression***<br><br>***In case of comorbidity: follow up necessary lab as needed***	HRCT of the chest<br><br>***Only repeat if there is symptomatic progression***	Baseline ECG (QTc is considered prolonged if greater than 450 ms in males and 470 ms in females).<br><br>***Only if the patient will receive azithromycin and/or hydroxychloroquine***
**Moderate cases**	•CBC, CRP, D-dimer, Ferritin, Liver function tests (ALT – AST) and kidney function tests (S.cr- BUN).	HRCT chest	Baseline ECG (QTc is considered prolonged if greater than 450 ms in

	- *Base line.*<br>- *Only abnormal findings are required to be repeated as needed*<br>*In case of comorbidity: follow up necessary lab as needed*	*Repeat if there is symptomatic progression and after 2 weeks of discharge*	males and 470 ms in females).<br>*Repeat every other day in patients receiving azithromycin and/ or hydroxychloroquine*
**Severe cases**	•CBC, CRP, D-dimer, Ferritin, Liver function tests (ALT – AST) and kidney function tests (S.cr-BUN).<br>- *Base line.*<br>- *Only abnormal findings are required to be repeated as needed*<br>*In case of comorbidity: follow up necessary lab as needed*	HRCT chest<br>*Repeat if there is symptomatic progression and after 2 weeks of discharge*	Baseline ECG (QTc is considered prolonged if greater than 450 ms in males and 470 ms in females).<br>*Repeat every other day in patients receiving azithromycin and hydroxychloroquine*
**Critical cases**	• CBC with differential, Urea/Electrolytes, Creatinine, CRP, LFTs, Ferritin, D-dimer, procalcitonin, Triglycerides, Troponin, creatine kinase, blood glucose, cultures , ECG, LDH.<br>- *Base line.*<br>- *Required lab are to be repeated as needed*<br>*In case of comorbidity: follow up necessary lab as needed*	HRCT chest<br>*Repeat if there is symptomatic progression and after 2 weeks of discharge*	Baseline ECG (QTc is considered prolonged if greater than 450 ms in males and 470 ms in females).<br>*ECG is to be repeated as needed*<br>*ECHO as needed*

This is an updatable dynamic statement based on current available information and clinical experience
