# Supplementary material for: Association between single nucleotide polymorphism of human angiotensin-converting enzyme 2 gene locus and clinical severity of COVID-19
Source: Egypt J Med Hum Genet. 2022 Aug 23;23(1):125. doi: 10.1186/s43042-022-00331-8 (PMC9395935; doi:10.1186/s43042-022-00331-8)
Supplement: Supplementary file 2 — Additional file 2. Table S1: Laboratory data among studied patients’ groups. Table S2a: Univariate logistic regression analysis for predictors of disease severity. Table S2b: Multivariate logistic regression analysis (Backward: Wald) for predictors of classification. [file 43042_2022_331_MOESM2_ESM.docx]

| **Laboratory data** | | **Moderate** | **Severe** | **Test value** | **P-value** | **Sig.** |
| --- | --- | --- | --- | --- | --- | --- |
|  |  | **No. = 19** | **No. = 71** |  |  |  |
| TLC n(x10^3 /UL) | Mean ± SD | 11.27 ± 4.33 | 16.45 ± 5.65 | -3.710• | 0.000 | HS |
|  | Range | 6 – 19 | 6.5 – 26 |  |  |  |
| Lymphocytic count  (x10^3 /UL) | Mean ± SD | 1.28 ± 0.58 | 0.86 ± 0.26 | 4.651• | 0.000 | HS |
|  | Range | 0.8 – 2.9 | 0.45 – 1.9 |  |  |  |
| Lymphocytic count | Normal | 9 (47.4%) | 8 (11.3%) | 12.750* | 0.000 | HS |
|  | Low | 10 (52.6%) | 63 (88.7%) |  |  |  |
| CRP (mg/dl) | Median (IQR) | 9 (7 – 9) | 49 (40 – 58) | -6.492 | 0.000 | HS |
|  | Range | 6 – 16 | 9 – 315 |  |  |  |
| Ferritin (ng/ml) | Median (IQR) | 259 (161 – 365) | 480 (411 – 650) | -5.671≠ | 0.000 | HS |
|  | Range | 155 – 401 | 160 – 1651 |  |  |  |
| D-dimer (mg/dl) | Median (IQR) | 1 (0.5 – 1.2) | 3.6 (1.9 – 5.10) | -5.303 | 0.000 | HS |
|  | Range | 0.3 – 6 | 0.6 – 12.00 |  |  |  |
| ALT, AST (U/L) | Mean ± SD | 28.42 ± 5.19 | 36.71 ± 8.02 | -4.262• | 0.000 | HS |
|  | Range | 21 – 38 | 20 – 61 |  |  |  |
| Serum creatinine  (mg/dL) | Median (IQR) | 1.1 (0.9 – 1.4) | 1.9 (1.4 – 5) | -4.640≠ | 0.000 | HS |
|  | Range | 0.8 – 1.7 | 1 – 13 |  |  |  |

Supplementary Table 1: Laboratory data among studied patients’ group.

TLC: Total leucocytic count, ALT: Alanine Aminotransferase, AST: Aspartate aminotransferase

*: Chi-square test; •: Independent t-test; ≠: Mann-Whitney test

Supplementary Table 2 a: Univariate logistic regression analysis for predictors of disease severity .

|  | **B** | **S.E.** | **Wald** | **P-value** | **Odds ratio  (OR)** | **95% C.I. for OR** | |
| --- | --- | --- | --- | --- | --- | --- | --- |
|  |  |  |  |  |  | **Lower** | **Upper** |
| HTN | 1.914 | 0.572 | 11.182 | **0.001** | 6.778 | 2.208 | 20.807 |
| Asthma | -2.575 | 1.187 | 4.700 | **0.030** | 0.076 | 0.007 | 0.781 |
| Anti-interleukin 6 (actemra) | 1.592 | 0.787 | 4.089 | **0.043** | 4.911 | 1.050 | 22.969 |
| Sepsis | 1.088 | 0.536 | 4.123 | **0.042** | 2.967 | 1.039 | 8.477 |
| TLC n(x10^3 /UL) | 2.044 | 0.674 | 9.203 | **0.002** | 7.724 | 2.062 | 28.938 |
| Low lymphocytic count | 1.958 | 0.593 | 10.896 | **0.001** | 7.087 | 2.216 | 22.672 |
| High ferritin | 4.587 | 1.078 | 18.088 | **0.000** | 98.182 | 11.858 | 812.905 |
| D-dimer (mg/dl) | 3.140 | 0.653 | 23.156 | **0.000** | 23.111 | 6.432 | 83.045 |
| High ALT, AST | 3.076 | 0.793 | 15.058 | **0.000** | 21.675 | 4.583 | 102.503 |
| High serum creatinine | 1.932 | 0.615 | 9.859 | **0.002** | 6.900 | 2.066 | 23.039 |
| Duration of hospital stay (days) | 2.056 | 0.784 | 6.867 | **0.009** | 7.811 | 1.679 | 36.338 |
| Genotype (rs 2048683) (GG/G) | 1.283 | 0.316 | 16.519 | **0.000** | 3.607 | 1.943 | 6.695 |

HTN:Hypertension

Supplementary Table 2 b: Multivariate logistic regression analysis (Backward: Wald) for predictors of classification.

|  | **B** | **S.E.** | **Wald** | **P-value** | **Odds ratio  (OR)** | **95% C.I. for OR** | |
| --- | --- | --- | --- | --- | --- | --- | --- |
|  |  |  |  |  |  | **Lower** | **Upper** |
| HTN | 1.948 | 1.058 | 3.388 | 0.066 | 7.012 | 0.882 | 55.773 |
| High ferritin | 5.091 | 1.530 | 11.076 | **0.001** | 162.589 | 8.108 | 3260.293 |
| Genotype (rs 2048683) (GG/G) | 1.767 | 0.666 | 7.036 | **0.008** | 5.852 | 1.586 | 21.591 |
| Duration of hospital stay (days) | 2.634 | 1.348 | 3.816 | 0.051 | 13.926 | 0.991 | 195.674 |

a Variable(s) entered on step 1: HTN, Asthma, Anti-interleukin 6 (actemra), Sepsis, TLC, Lymphocytic count, Ferritin, D-dimer, ALT, AST, Serum creatinine, Duration of hospital stay (days) and Genotype (rs 2048683).
